# Immunomodulatory Effect of *Artemisia annua* L. Water Extract on Meat-Type Sheep via Activating TLR4/NF-κB Signaling Pathways

**DOI:** 10.3390/ani16010059

**Published:** 2025-12-24

**Authors:** Gen Gang, Ruiheng Gao, Shiwei Guo, Yu Xin, Xiao Jin, Yuanyuan Xing, Sumei Yan, Yuanqing Xu, Binlin Shi

**Affiliations:** 1 College of Animal Science, Inner Mongolia Agricultural University, Hohhot 010018, China; ganggen@emails.imau.edu.cn (G.G.); gaoruiheng@emails.imau.edu.cn (R.G.); guoshiwei@emails.imau.edu.cn (S.G.); yaojinxiao@imau.edu.cn (X.J.); xingyuanyuan2014@163.com (Y.X.); yansmimau@imau.edu.cn (S.Y.); 2 National Center of Pratacultural Technology Innovation (Under Preparation), Hohhot 010060, China; yuxin119822@163.com

**Keywords:** water extracts of *Artemisia annua* L., immunomodulatory effect, TLR4/NFκB pathway, peripheral blood lymphocyte, meat-type sheep

## Abstract

This experiment was designed to systematically evaluate the immunomodulatory effect of water extract of *Artemisia annua* L. (WEAA) on sheep, both in vivo and in vitro, and to determine the involvement of the TLR4/NF-κB signaling pathway in mediating these effects. In experiment 1, 32 female sheep (Dorper × Han, 3 months old, 24 ± 0.09 kg each) were designated to 4 groups, with each group receiving a basal diet supplemented with, respectively, 0 (control group), 500, 1000, and 1500 mg/kg WEAA. The serum, liver, and spleen immune indicators and related gene expressions were measured. In experiment 2, the peripheral blood lymphocytes (PBLs) were processed with WEAA (0, 25, 50, 100, 200, and 400 μg/mL), with six replicates assigned to each concentration group, then the cell viability, immune function, and related gene expressions were measured, and the optimal concentration of WEAA was determined. In experiment 3, the experimental groups consisted of PBLs subjected to treatments with or without PDTC (NF-κB inhibitor) and with or without WEAA, forming four distinct treatment groups (six replicates/group): PDTC(−)/WEAA(−) group, PDTC(−)/WEAA(+) group, PDTC(+)/WEAA(−) group and PDTC(+)/WEAA(+) group. The immune indexes and TLR4/NFκB pathway related indexes were determined. The results were as follows: WEAA dose-dependently enhanced the content of immunoglobulins (IgA, IgG, IgM) and cytokines (IL-1β, IL-2, IL-4) in the serum, liver, and spleen tissues, among which IgA, IgG, and IL-4 were the most significantly affected core indicators (*p* < 0.05). Meanwhile, WEAA dose-dependently upregulated the expression of TLR4/NF-κB pathway-related genes (*TLR4*, *IKKβ*, *IκBα*, *NF-κBp65*) and their downstream cytokine-related genes (*IL-1β*, *IL-4*) in liver and spleen tissues (*p* < 0.05). Of these genes, liver *IL-4*, *IκBα*, and spleen *IL-4* were the most prominently regulated core genes (*p* < 0.05), The optimal supplementary dose of WEAA was determined to be 1000 mg/kg. In addition, adding 100 μg/mL WEAA to the culture medium of PBLs significantly enhanced immune function and cell viability. The underlying mechanism involved the TLR4/NF-κB pathway; that is to say, WEAA enhanced sheep’s immune indicators by upregulating TLR4/NF-κB pathway genes, thereby coordinately regulating humoral and innate immunity, thereby improving the immune indices of sheep. This study provided compelling experimental support for the prospective utilization of WEAA as a functional feed supplement in intensive meat-type sheep production systems.

## Simple Summary

Due to the rapid advancement of intensive meat-type sheep farming in China, the industry is confronting a series of stress challenges, including high-density rearing environments, environmental pollution, imbalanced feed nutrition, and frequent disease outbreaks. These stressors may lead to oxidative imbalance and immune dysfunction in sheep, thereby adversely affecting livestock health and production performance. *Artemisia annua* L. and its extracts contain various bioactive compounds and exhibit immunomodulatory effects, thereby potentially enhancing the health status of animal organisms. Based on the abovementioned potential, this study aimed to comprehensively evaluate the immunomodulatory function of water extract of *Artemisia annua* L. (WEAA) in the serum, liver, and spleen of sheep and explore the underlying mechanistic pathways via in vivo and in vitro experiments. The results showed that dietary supplementation of WEAA enhanced sheep’s immune indicators by upregulating TLR4/NF-κB pathway genes, thereby coordinately regulating humoral and innate immunity, thereby improving the immune indices of sheep.

## 1. Introduction

The global demand for animal protein, particularly from sheep, continues to rise, placing considerable pressure on production systems to enhance efficiency and output [1,2]. Concurrently, challenges such as land degradation and the industry’s pursuit of improving market competitiveness of livestock products have intensified [3]. Intensive meat-type sheep farming, characterized by high stocking densities and optimized production cycles, has become a cornerstone of this supply chain. However, such intensification often brings forth a spectrum of immunological challenges. Animals in these systems can experience heightened stress levels due to confinement, social interactions, and management practices, which can compromise their immune competence, rendering them more susceptible to a variety of pathogens and metabolic disorders [4]. The increased risk of disease transmission in densely populated environments necessitates robust immune function for maintaining animal health and welfare, and for ensuring the consistent productivity of farming operations [5,6]. Therefore, interest in optimizing dietary nutrition strategies to strengthen sheep health and productivity has grown. Among such strategies, naturally sourced feed supplements have distinguished themselves as promising substitutes for synthetic growth stimulants and antibiotics in livestock production systems [7], owing to their diverse biological functionalities (including antibacterial, antioxidant, and gut microbiota-regulating properties), low risk of inducing drug resistance, minimal residue concerns, abundant plant-derived resources, and alignment with green agriculture trends [8]. Recent studies have underscored the capacity of *Artemisia* species and their extracts to positively regulate immune function across diverse animal models. Specifically, experimental studies have specifically elucidated the immunomodulatory effects of these botanical phytochemicals on the immune systems of broiler chickens, piglets, and mice [9,10,11]. For instance, dietary supplementation with *Artemisia argyi* (*A. argyi*) flavonoids has been demonstrated to improve immune function in LPS-challenged broilers, as evidenced by the suppressed overexpression of *IL-1β* and *IL-6*, as well as the inhibited hyperactivation of the TLR4-NFκB signaling pathway, in serum, liver, and spleen tissues [12]. Similarly, *A. ordosica* and its bioactive extracts (polysaccharides and flavonoids) were also found to modulate the immune function of broilers and mice [13,14].

*Artemisia annua* L. (*A. annua*) has been traditionally used as an herbal remedy for malaria in China [15,16]. Notably, this plant is rich in bioactive constituents, including polysaccharides, flavonoids (e.g., luteolin, rutin, kaempferol, quercetin, centaureidin), terpenoids (e.g., artemisinin, abscisic acid, perilla alcohol, arteannuin B), coumarins, and essential oils. These compounds have been proven to exhibit antibacterial [17,18], anti-inflammatory, anti-tumor, and antioxidant effects [19,20], and is thought to act synergistically to produce a broader range of therapeutic effects than any single isolated constituent [21]. A study showed that dietary addition of *A. annua* for pigs in the late gestational and lactational phases boosted the intestinal barrier function of their offspring [22]. In our previous study, dietary supplementation with WEAA was also found to regulate immune status and antioxidant capacity in broilers, as evidenced by enhanced immunoglobulin levels (IgM, sIgA) and increased activity of antioxidant enzymes (CAT, SOD, GPx)—changes that correlate with enhanced immune status [23]. However, previous research on WEAA mainly focused on monogastric animals (such as pigs, broilers, and rats). Given the fundamental differences in digestive and metabolic physiology between ruminants and monogastric animals, findings from monogastric studies cannot be extrapolated to ruminants, highlighting an urgent need for targeted research on WEAA in meat sheep. To date, systematic studies on its immunoregulatory effects and mechanisms in ruminants, particularly meat-type sheep, remain scarce. Furthermore, the underlying molecular mechanisms of plant extracts’ immunomodulatory effects are complex and involve regulating key intracellular signaling pathways. Among these, the NF-κB pathway serves as an essential regulator of immune function and inflammatory responses [24]. NF-κB is a family of transcription factors that undergoes nuclear translocation upon activation and subsequently initiates the transcriptional regulation of genes involved in immunoregulatory processes, inflammatory responses, cellular homeostasis, and mitotic progression. As a pattern recognition receptor, TLR4 can regulate NF-κB nuclear translocation via downstream signaling cascades. Regulating the NF-κB signaling pathway exerts protective effects against intestinal inflammation in weaned piglets [25], as well as *E. coli*-induced or LPS-induced inflammatory responses in broiler chickens or lambs [26,27]. Studies have shown that plant polysaccharides act as TLR4 ligands, directly binding to the TLR4/MD2 complex on immune cells (e.g., macrophages, lymphocytes). This interaction recruits downstream adapter proteins, triggering NF-κB pathway activation. Consequently, it promotes transcription and secretion of key cytokines, ultimately boosting humoral and cellular immunity [28]. Secondary metabolites (e.g., terpenoids and flavonoids) possess antioxidant properties that scavenge ROS generated during pathway activation, thereby preventing oxidative damage to immune cells. Additionally, terpenoids (e.g., artemisinin B, cembrane diterpenoids, euphorbia factor L2) exert multi-targeted regulation of NF-κB transcriptional activity through three key mechanisms: inhibiting the phosphorylation of inhibitor of IKKα/β, reducing K63-linked ubiquitination of tumor necrosis factor receptor-associated factor 6 (TRAF6), and suppressing acetylation modification of NF-κB p65 [29]. Flavonoids (e.g., cyanidin, quercetin, kaempferol) upregulate the expression of anti-inflammatory cytokine, restoring balance with pro-inflammatory cytokines [30]. Notably, active components in *A. annua* have shown immunomodulatory potential. For example, Zhang et al. (2022) [31] found that *A. annua* polysaccharides significantly increased the levels of the pro-inflammatory cytokines IL-6 and TNF-α in murine macrophages in a dose-dependent manner, exhibiting low cytotoxicity. Similarly, studies have demonstrated that dietary inclusion of *A. ordosica* polysaccharides in broilers can effectively activate the downstream signaling of the TLR4/MAPK/NF-κB pathway. This activation upregulates the expression of key molecules (e.g., *TLR4*, *MyD88*, *MAPK*, *NF-κB*, *IL-1β*), thereby balancing the secretion of pro-inflammatory cytokines (e.g., IL-1β, IL-6, TNF-α) and anti-inflammatory cytokines (e.g., IL-2, IL-4), which subsequently enhanced immune competence and disease resistance in broilers [13]. The literature has established that plant polysaccharides can specifically bind to pattern recognition receptors such as TLR2/4, initiating downstream signaling pathway cascades [12]. This evidence provides a molecular basis for the association between *A. annua* bioactive constituents and the TLR4/NF-κB pathway. As core effector cells of the adaptive immune system, peripheral blood lymphocytes (PBLs) directly participate in antigen recognition, immune signal transduction, and cytokine secretion. The functional activity of these cells is a key indicator reflecting the body’s immune status [32]. Moreover, PBLs express high levels of TLR4 receptors on their surface, and their immune functions are closely associated with the activity of the TLR4/NF-κB signaling pathway [33]. Additionally, PBLs offer advantages such as convenient sampling and high stability in in vitro culture, enabling precise control of variables like WEAA concentration during experiments, which complements in vivo studies. Therefore, PBLs were chosen to serve as the cellular model for elucidating the molecular mechanisms in this study. The water extraction process of *A. annua* effectively preserves its water-soluble components (such as polysaccharides, flavonoids, terpenes, and phenolics), which act synergistically and exhibit multiple immune-regulatory beneficial biological activities, such as antimicrobial, anti-inflammatory, antioxidative, and immunomodulatory effects. Given that TLR4/NF-κB is a classical immune regulatory pathway, whether the immunomodulatory effect of WEAA is mediated through this pathway needs to be investigated further. This study used meat-type sheep and their PBLs as research subjects, aiming to systematically investigate the effect of dietary supplementation with WEAA on the immunoregulatory function of meat-type sheep. It focuses on clarifying whether WEAA exerts its effect by regulating the TLR4/NF-κB signaling pathway, thereby providing theoretical support for the application of WEAA in green breeding practices for meat-type sheep.

## 2. Materials and Methods

### 2.1. Preparation of Water Extract of Artemisia annua L. (WEAA)

*A. annua* was harvested in Hohhot, Inner Mongolia. Its fresh aerial parts were collected and shade-dried and cut into small fragments (approximately 0.5–1.5 cm in length) to increase the contact surface area. The above pre-extraction pretreatment and subsequent process parameters were determined based on the detailed protocol of Guo et al. [23] combined with preliminary optimization, and the specific procedures were as follows: the dried plant segments were weighed precisely and mixed with distilled water at a solid-to-solvent ratio of 1:25 (*w*/*v*) and then heated in a water bath at 80 ± 5 °C for 7 h. After completion of the extraction, the mixture underwent vacuum filtration to separate the solution from the solid residues. This solution was concentrated under reduced pressure using a rotary evaporator (Shanghai Yarong Rotary Evaporator RE-5298 A, Shanghai, China) at 80 °C until a viscous concentrate was obtained. The concentrate was then lyophilized (freeze-dried) using a freeze dryer (ALPHA 1–2 LD plus, Martin Christ Gefriertrocknungsanlagen GmbH, Osterode am Harz, Germany) at −40 °C and 0.1 mbar for 48 h to produce a dry powder. All extracts were stored at 4 °C, pending subsequent analysis or experimental application.

Metabolite identification of WEAA was performed using UPLC-Orbitrap-MS technology. After raw data were preprocessed with Progenesis QI 2.4 software (including baseline filtering, peak matching, retention time calibration, etc.), metabolite matching was conducted by combining commercial databases with the self-established metabolite MS^2^ database of Jiangsu Sanshu Biotechnology Co., Ltd. (Nantong, China), where a secondary fragment score > 0.7 was considered reliable identification. Further reliability screening was implemented based on the following criteria: molecular mass error < 5 ppm, relative standard deviation (RSD) of parallel experiments < 10%, and exclusion of uncharacterized “Unknown” substances. Finally, major metabolites, including organic acids (24.61%), polysaccharides (18.64%), flavonoids (9.80%), and prenol lipids (7.75%), were detected. Among these, the inter-batch RSD of key metabolites was <10%, and the proportion of core components remained stable. This indicated that WEAA possessed good stability in terms of chemical composition and active component content, meeting the quality requirements for experimental samples. For details, please refer to Supplementary Table S1, Figures S1 and S2.

### 2.2. Animals, Experimental Design, and Management Protocols

All experimental animals were procured from a livestock farm located in Ordos City, Inner Mongolia Autonomous Region, China, and were confirmed to be in good health following a comprehensive pre-experiment health evaluation. All experiments involving the animals adhered to the Health Guidelines for the Care and Use of Laboratory Animals and the experiment was authorized by Inner Mongolia Agricultural University’s Institutional Ethics Committee (No. NND2021097), conducted in full compliance with the National Standard Operating Procedures (GB/T 43562-2023, Laboratory animal-Guideline for ethical review of animal welfare, China) [34].

Thirty-two clinically healthy, 3-month-old female meat-type sheep (Dorper × Han sheep, initial body weight: 24 ± 0.09 kg) were designated and kept in standardized environmental conditions for seven days prior to trial initiation. During this acclimation period, animals had ad libitum access to a basal diet and fresh water, maintained under uniform environmental conditions. Employing a completely randomized block design, the sheep were allocated into four treatment groups (0, 500, 1000, 1500 mg/kg diet, respectively, *n* = 8), with the dose gradient determined by referencing prior studies reporting effective doses of *Artemisia* extracts in ruminant nutrition [35,36,37].

Sheep were tested for health status and dewormed prior to the experiment. The experiment lasted 75 days and was divided into two phases: a 15-day baseline phase (days 1–15) for diet adaptation and baseline data collection, followed by a 60-day formal trial period (days 16–75). The basal diet formulation strictly followed the nutritional specifications outlined in Nutrient Requirements of Meat-type Sheep (NY/T 816-2021) [38], consisting of 45% concentrate and 55% forage (alfalfa hay, corn straw, and oat grass). Detailed compositional analysis and nutritional profiles are provided in Supplementary Table S2. Each sheep was housed individually in pens measuring 2.4 m × 3.6 m and provided pelleted feed ad libitum in two daily meals (08:00 and 16:00); fresh water was available at all times for free access. Daily feed intake was recorded to determine the average daily feed intake (ADFI). The feed supply was adjusted daily to maintain 5% orts. Body weight was measured on days 0, 30, and 60 of the experiment before the morning feeding to calculate the average daily gain (ADG) and feed conversion ratio (F/G).

### 2.3. Sample Collection

On experimental days 0, 30, and 60, 10 mL of whole blood was harvested via jugular venipuncture in the morning. Samples were centrifuged to isolate serum, then cryopreserved at −20 °C for subsequent quantification of immunoglobulins (IgG, IgA, IgM) and cytokines (IL-2, IL-4, IL-1β, IL-6).

Upon termination of the trial period, liver and spleen samples were collected from slaughtered sheep. Around 1 g of liver and spleen tissue from each organ was immediately rinsed with physiological saline, snap-frozen in liquid nitrogen, and ultimately stored at −80 °C for later analysis.

### 2.4. Cell Culture

Peripheral venous blood was obtained from 3 healthy female Dorper × Han sheep (3 months old, 26 ± 3.5 kg) via jugular venipuncture using heparinized syringes (10 IU heparin/mL blood). Prior to sampling, the sheep were fasted for 10 h, with ad libitum access to water. The blood sample was diluted at a 1:1 ratio with sterile PBS (pH 7.2–7.4) and gently overlaid onto sheep lymphocyte separation medium equal to that of the diluted blood in 15 mL centrifuge tubes, ensuring a sharp interface between the two phases. Density gradient centrifugation was performed at 375× *g* for 20 min at room temperature without applying the brake. The buffy coat containing lymphocytes was harvested and washed twice with PBS (300× *g*, 10 min, 4 °C) to eliminate residual separation medium. Isolated PBLs were resuspended in RPMI-1640 culture medium supplemented with 10% heat-inactivated fetal bovine serum (FBS), 100 U/mL penicillin, and 100 μg/mL streptomycin. Using an automated cell counter, the concentration of viable PBLs was adjusted to 1 × 10^6^ cells/mL, and the suspension was plated into 24-well plates (1.8 mL per well) under sterile conditions in a laminar flow hood.

### 2.5. Cell Viability Assay and Treatment

PBLs were allocated into 6 treatment groups (6 replicates each) with supplementation of WEAA at final concentrations of 0, 25, 50, 100, 200, and 400 μg/mL, respectively. The culture volume in each well was adjusted to 2 mL with complete RPMI-1640 medium, followed by incubation at 37 °C in a 5% CO_2_ humidified incubator for 24 h. For viability assessment, 100 μL of cell suspension from each well was transferred to a 96-well plate, and 10 μL of CCK-8 reagent was added. After 3 h of incubation, absorbance at 450 nm was measured using a microplate reader to calculate cell viability. Remaining cultures were centrifuged (210× *g*, 10 min, 4 °C); supernatants were stored at −20 °C for analyzing the content of immunoglobulins and cytokines, while cells were frozen at −80 °C for subsequent RNA extraction and gene expression analysis.

### 2.6. Treatment of PBLs with Signaling Inhibitors

A 2 × 2 factorial design was employed to explore the role of the TLR4/NFκB pathway, involving two factors: PDTC (NFκB inhibitor) and WEAA, each with two levels (presence or absence) [39,40]. Four treatment groups (6 replicates each) were set for 24.5 h culture as follows:

Control group [PDTC(−)/WEAA(−)]: Cultured in complete medium without any treatment.

WEAA-treated group [PDTC(−)/WEAA(+)]: Equilibrated for 0.5 h, then treated with 100 μg/mL WEAA for 24 h.

PDTC-treated group [PDTC(+)/WEAA(−)]: Treated with 10 μmol/L PDTC (NF-κB inhibitor) at 0 h, maintained throughout the 24.5 h culture.

Combined group [PDTC(+)/WEAA(+)]: Pre-treated with 10 μmol/L PDTC for 0.5 h, then co-cultured with 100 μg/mL WEAA for 24 h (PDTC maintained at 10 μmol/L).

### 2.7. Immunoglobulins and Cytokines

The content of immunoglobulins and interleukins were measured using commercial ELISA kits in accordance with the manufacturer’s instructions.

### 2.8. qRT-PCR Analysis

Total RNA was extracted from spleen tissue using TRIzol reagent, with integrity and purity verified before selecting qualified samples (OD260/OD280: 1.8–2.1) for cDNA synthesis. The resulting cDNA was stored at −20 °C for later use. qPCR reactions were conducted on a Light Cycler^®^ 96 real-time quantitative PCR instrument using SYBR Green Master Mix (No Rox) with cDNA as the template. Refer to our previously published article for detailed reaction protocols [41]. *β-actin* and *GAPDH* were employed as internal reference genes, and detailed primer information is provided in Supplementary Table S3. The relative expression levels of target genes were calculated using the 2^−ΔΔCt^ method.

### 2.9. Statistical Analysis

All data were initially organized and preprocessed using Microsoft Excel 2021. Linear and quadratic regression analyses were performed with SAS 9.4 software to evaluate the relationships between increasing WEAA concentrations and various response variables. For the 2 × 2 factorial design (with PDTC and WEAA as independent factors), ANOVA was applied to examine the main effects of each factor and their interaction. Duncan’s multiple range test was used for post hoc comparisons between groups. The results are presented as the mean ± SEM, with *p* ≤ 0.05 (significant), *p* < 0.01 (highly significant), and 0.05 < *p* < 0.10 (tendency toward significance).

## 3. Results

### 3.1. Growth Performance

As shown in Table 1, in the early stage of the experiment, diets supplemented with 1000 mg/kg WEAA decreased ADFI (*p* = 0.05) and all WEAA supplementation lowered the F/G compared with the control group (*p* < 0.05). Furthermore, with the increase in WEAA dosage, both ADFI and F/G exhibited a linear or quadratic reduction (*p* < 0.05). In the later stage of the experiment, diets containing 500 and 1000 mg/kg WEAA significantly lowered ADFI compared to the control group (*p* < 0.05), and the ADFI exhibited a linear decreasing trend (*p* = 0.065) or a significant quadratic decrease (*p* < 0.05). Throughout the entire experiment, WEAA supplementation decreased ADFI compared to the control group (*p* = 0.05), and the ADFI exhibited a linear decreasing trend (*p* = 0.050) or a significant quadratic decrease (*p* < 0.05). Additionally, diets containing 1000 mg/kg WEAA decreased F/G (*p* < 0.05), while the F/G exhibited a quadratic decreasing trend (*p* = 0.050).

### 3.2. Immune Indices in Sheep

**Figure 1:**
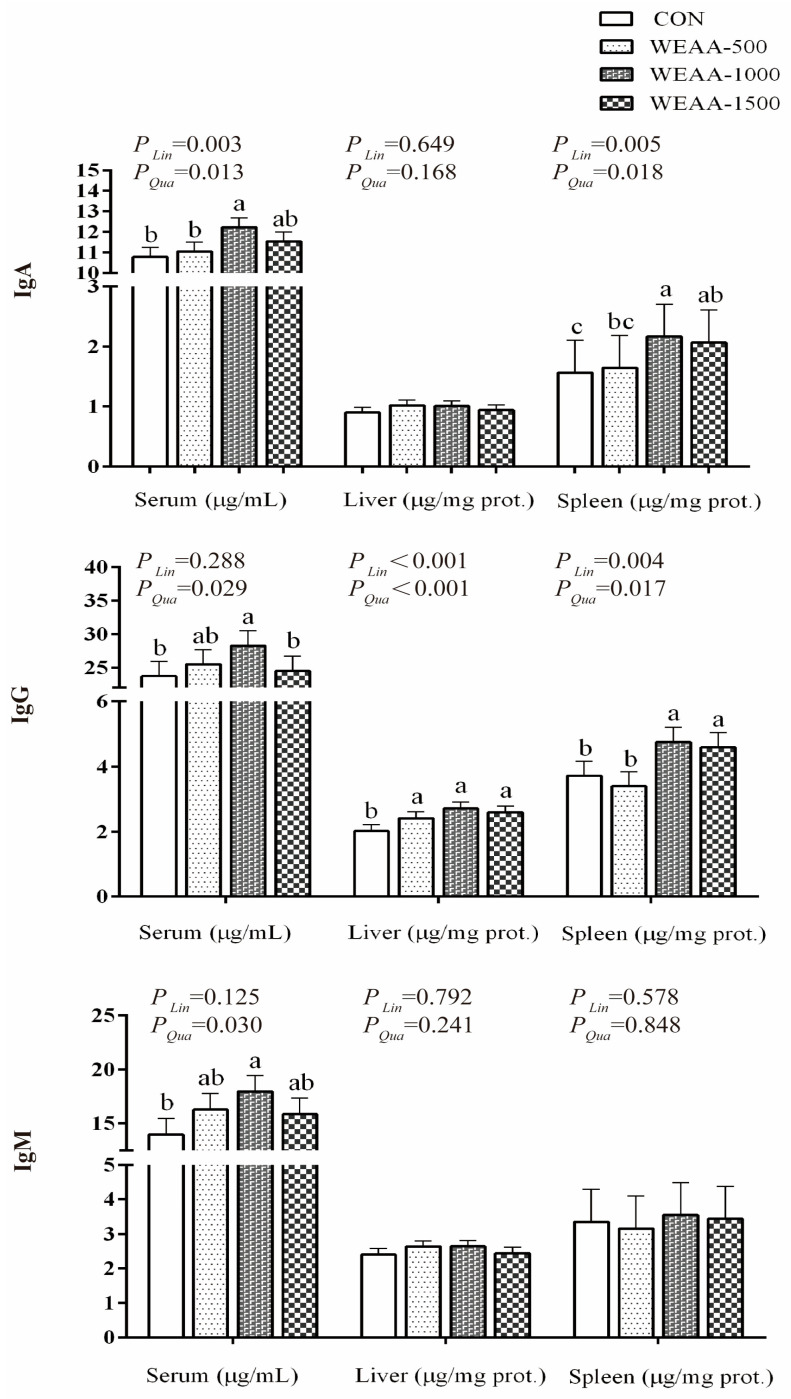
Effects of dietary WEAA on the level of immunoglobulins in meat-type sheep. Abbreviations: IgA = Immunoglobulin A; IgG = Immunoglobulin G; IgM = Immunoglobulin M; WEAA = Water Extracts of *Artemisia annua* L.; CON = Control; WEAA-500 = 500 mg/kg WEAA; WEAA-1000 = 1000 mg/kg WEAA; WEAA-1500 = 1500 mg/kg WEAA; values are expressed as the means of 8 sheep in each group. Dose-dependent effects of WEAA (Lin = Linear; Qua = Quadratic), assesses the linear and quadratic effects on various response indices as a function of increasing WEAA supplementation levels. Values within a row with different superscripts (a, b, c) differ significantly at *p* ≤ 0.05, whereas the probability value of 0.05 < *p* < 0.10 was considered as a tendency.

Figure 1 illustrates the immunoglobulin content in serum, liver, and spleen tissues. Compared with the control group, diets supplemented with WEAA at a dose of 1000 mg/kg significantly increased the IgA content in serum (*p* < 0.05), and 1000 and 1500 mg/kg WEAA significantly increased the IgA content in the spleen (*p* < 0.05). With increasing dietary WEAA levels, the IgA levels in serum and spleen demonstrated a significant linear or quadratic increase (*p* < 0.01; *p* < 0.05; *p* < 0.01; *p* < 0.05). Additionally, diets containing 1000 mg/kg WEAA elevated the serum IgG content (*p* < 0.05), and with the increase in WEAA dosage, the serum IgG levels showed a significant quadratic increase (*p* < 0.05). Compared with the control group, WEAA supplementation significantly increased the liver IgG levels (*p* < 0.05), while 1000 and 1500 mg/kg WEAA significantly elevated the spleen IgG levels (*p* < 0.05). The IgG levels in liver and spleen exhibited a significant linear or quadratic increase (*p* < 0.01; *p* < 0.01; *p* < 0.01; *p* < 0.05). Moreover, the inclusion of 1000 mg/kg WEAA significantly increased the serum IgM content compared to the control group (*p* < 0.05), and the serum IgM levels also demonstrated a significant quadratic increase (*p* < 0.05).

**Figure 2:**
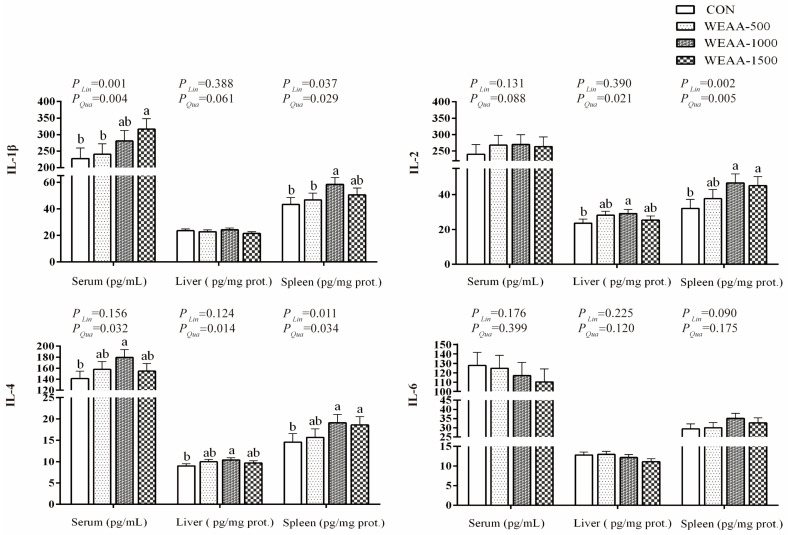
Effects of dietary WEAA on content of cytokines in meat-type sheep. Abbreviations: IL-1β = Interleukin 1β; IL-2 = Interleukin 2; IL-4 = Interleukin 4; IL-6 = Interleukin 6; WEAA = Water Extracts of *Artemisia annua* L.; CON = Control; WEAA-500 = 500 mg/kg WEAA; WEAA-1000 = 1000 mg/kg WEAA; WEAA-1500 = 1500 mg/kg WEAA; values are expressed as the means of 8 sheep in each group. Dose-dependent effects of WEAA (Lin = Linear; Qua = Quadratic), assesses the linear and quadratic effects on various response indices as a function of increasing WEAA supplementation levels. Values within a row with different superscripts (a, b) differ significantly at *p* ≤ 0.05, whereas the probability value of 0.05 < *p* < 0.10 was considered as a tendency.

In Figure 2, compared with the control group, the diet containing 1500 mg/kg WEAA increased the serum IL-1β levels (*p* < 0.05), and 1000 mg/kg WEAA significantly elevated the spleen IL-1β levels (*p* < 0.05), while the serum and spleen IL-1β levels showed a linear or quadratic increase (*p* < 0.01; *p* < 0.01; *p* < 0.05; *p* < 0.05). Compared with the control group, the diet with 1000 mg/kg WEAA increased the liver IL-2 levels (*p* < 0.05), and 1000 and 1500 mg/kg WEAA significantly increased the spleen IL-2 levels (*p* < 0.05). With the increase in WEAA dose, the serum concentrations of IL-2 showed a quadratic upward trend (*p* = 0.088) and the liver IL-2 levels exhibited a quadratic increase (*p* < 0.05), while the spleen IL-2 levels showed a significant linear or quadratic increase (*p* < 0.05). Additionally, compared with the control group, the diets supplemented with 1000 mg/kg WEAA increased the IL-4 levels in serum and liver (*p* < 0.05), and 1000 and 1500 mg/kg WEAA significantly elevated the spleen IL-4 levels (*p* < 0.05). With the increase in WEAA dose, the serum and liver concentrations of IL-4 demonstrated quadratic increases (*p* < 0.05) and the spleen IL-4 levels exhibited a linear or quadratic increase as WEAA dosage rose (*p* < 0.05).

### 3.3. mRNA Expression in the Liver and Spleen

**Figure 3:**
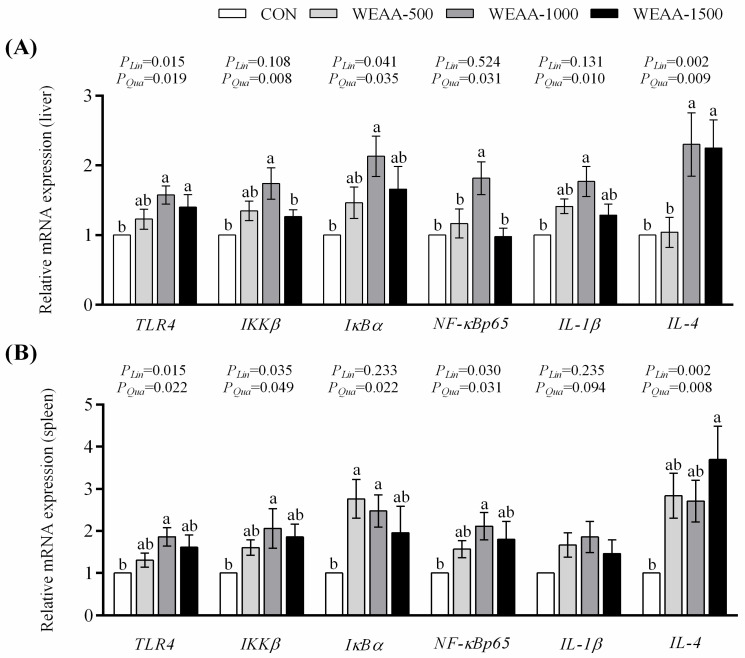
(**A**) Effect of dietary WEAA levels on the expression of TLR4/NF-κB signaling pathway-related gene in the liver of meat-type sheep. (**B**) Effect of dietary WEAA levels on the expression of TLR4/NF-κB signaling pathway-related gene in the spleen of meat-type sheep. Abbreviations: TLR4 = Toll Like Receptor 4; IKKβ = Inhibitor of Nuclear Factor Kappa B Kinase Subunit Beta; IκB-α = NFKB Inhibitor Alpha; NFκBp65 = RELA Proto-Oncogene; IL-1β = Interleukin 1β; IL-4 = Interleukin 4; WEAA = Water Extracts of *Artemisia annua* L.; CON = Control; WEAA-500 = 500 mg/kg WEAA; WEAA-1000 = 1000 mg/kg WEAA; WEAA-1500 = 1500 mg/kg WEAA; values are expressed as the means of 8 sheep in each group. Dose-dependent effects of WEAA (Lin = Linear; Qua = Quadratic), assesses the linear and quadratic effects on various response indices as a function of increasing WEAA supplementation levels. Values within a row with different superscripts (a, b) differ significantly at *p* ≤ 0.05, whereas the probability value of 0.05 < *p* < 0.10 was considered as a tendency.

Figure 3A showed that dietary supplementation with 1000 and 1500 mg/kg WEAA enhanced the liver *TLR4*, and *IL-4* expression compared to the control group (*p* < 0.05), which demonstrated a significant linear or quadratic elevation effect with the increase in WEAA dose (*p* < 0.05). Diet containing 1000 mg/kg WEAA significantly enhanced *IKKβ*, *IκBα*, *NF-κBp65* and *IL-1β* expression in the liver compared to the control group (*p* < 0.05), and the expression of *IκBα* exhibited a linear or quadratic increase (*p* < 0.05), while *IKKβ*, *NF-κBp65* and *IL-1β* expression showed a quadratic increase in the liver with the increase in WEAA dose (*p* < 0.05).

Figure 3B showed that a diet with 1000 mg/kg WEAA increased *TLR4*, *IKKβ*, and *NF-κBp65* expression in the spleen compared to the control group (*p* < 0.05), which demonstrated a significant linear or quadratic increase with the increase in dietary WEAA supplementation (*p* < 0.05). Additionally, diet containing 500 and 1000 mg/kg WEAA exhibited an increase in the expression of *IκBα* compared to the control group (*p* < 0.05), which demonstrated a quadratic increase with the increase in WEAA supplementation (*p* < 0.05). A diet containing 1500 mg/kg WEAA also increased spleen *IL-4* expression (*p* < 0.01), which exhibited a significant linear or quadratic increase as WEAA dosage rose (*p* < 0.01).

### 3.4. Cell Viability of PBLs

**Figure 4:**
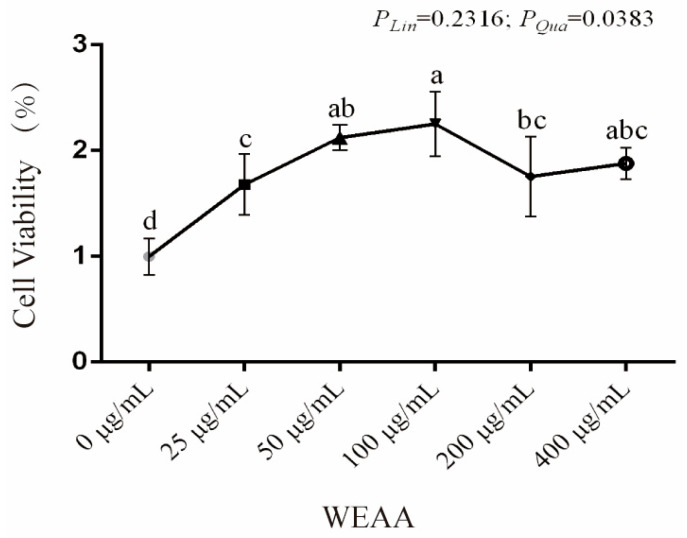
Effects of WEAA on cell viability in PBL (*n* = 6). Abbreviations: WEAA = Water Extracts of *Artemisia annua* L. Different concentrations of WEAA (0, 25, 50, 100, 200 and 400 µg/mL). Dose-dependent effects of WEAA (Lin = Linear; Qua = Quadratic), assesses the linear and quadratic effects on various response indices as a function of increasing WEAA supplementation levels. Values within a row with different superscripts (a, b, c) differ significantly at *p* ≤ 0.05, whereas the probability value of 0.05 < *p* < 0.10 was considered as a tendency.

As shown in Figure 4, with the increase in the addition level of WEAA, the viability of PBLs exhibited a significant quadratic increasing effect (*p* < 0.05). Among the tested concentrations, 100 μg/mL WEAA exhibited the most significant effect on cell viability; therefore, this concentration was selected for subsequent experiments.

### 3.5. Immune Indices and mRNA Expression in PBLs

Table 2 showed the effects of WEAA on immune function indices in PBLs culture medium. Compared with the control group, addition with 50–100 µg/mL WEAA enhanced the IgA level (*p* < 0.05), while 50–200 µg/mL WEAA elevated the IL-2 content in PBLs medium (*p* < 0.05). Both the IgA and IL-2 levels exhibited a quadratic increase with rising added WEAA concentrations (*p* < 0.05). Additionally, 50–400 µg/mL WEAA supplementation increased IgG and IL-4 levels in PBLs medium compared with the control group (*p* < 0.05), the IgG content showed a significant quadratic increase (*p* < 0.01), whereas IL-4 content displayed a linear increasing trend (*p* = 0.053) and a significant quadratic increase with higher WEAA doses (*p* < 0.01). Supplementation with 100 and 200 µg/mL WEAA increased the IgM level in PBLs culture medium compared to the control group (*p* < 0.05), with IgM content showing a quadratic increasing trend as added WEAA levels rose (*p* = 0.052). Moreover, 100–400 µg/mL WEAA increased IL-1β content compared to the control group (*p* < 0.05), and IL-1β content exhibited a linear or significant quadratic (*p* < 0.05; *p* < 0.01) increase with increasing WEAA dose.

**Figure 5:**
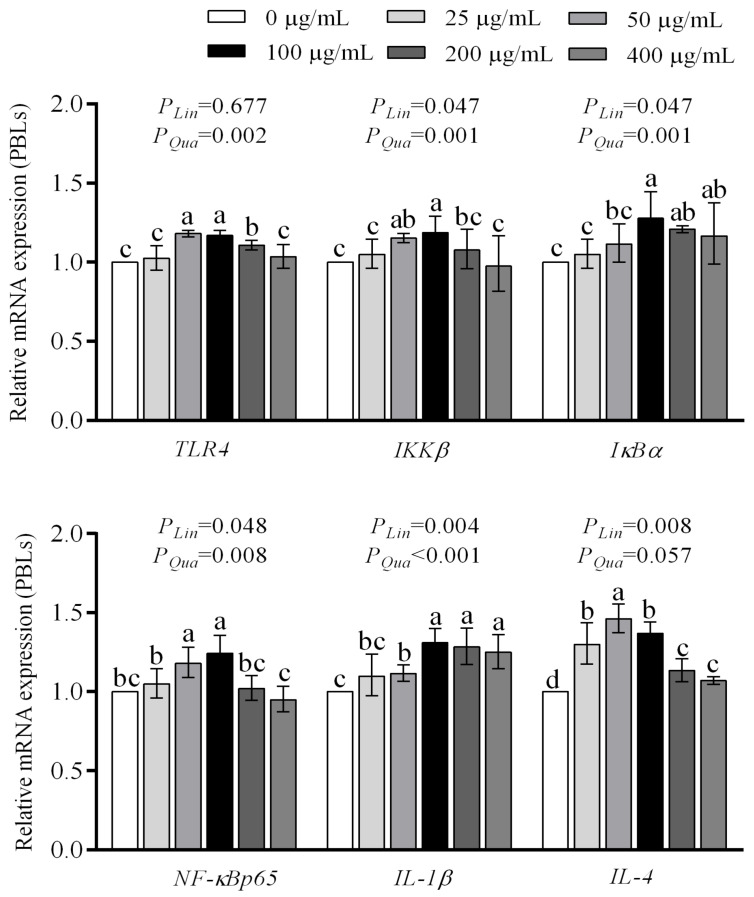
Effect of WEAA levels on the expression of TLR4/NF-κB signaling pathway-related genes in PBLs. Abbreviations: TLR4 = Toll Like Receptor 4; IKKβ = Inhibitor of Nuclear Factor Kappa B Kinase Subunit Beta; IκB-α = NFKB Inhibitor Alpha; NFκBp65 = RELA Proto-Oncogene; IL-1β = Interleukin 1β; IL-4 = Interleukin 4; WEAA = Water Extracts of *Artemisia annua* L. Different concentrations of WEAA (0, 25, 50, 100, 200 and 400 µg/mL), values were expressed as the means of 6 replicates in each group. Dose-dependent effects of WEAA (Lin = Linear; Qua = Quadratic), assesses the linear and quadratic effects on various response indices as a function of increasing WEAA supplementation levels. Values within a row with different superscripts (a, b, c) differ significantly at *p* ≤ 0.05, whereas the probability value of 0.05 < *p* < 0.10 was considered as a tendency.

Figure 5 showed that supplementation with 50–200 µg/mL WEAA increased the *TLR4* expression compared with the control group (*p* < 0.05), which demonstrated a significant quadratic increase (*p* < 0.01). Compared with the control group, supplementation with 50 and 100 µg/mL WEAA elevated the *IKKβ* and *NF-κBp65* expression (*p* < 0.05), and 100–400 µg/mL WEAA increased the *IκBα* expression (*p* < 0.05), and 50–400 µg/mL WEAA increased the *IL-1β* expression (*p* < 0.05), while all WEAA supplementation groups increased the *IL-4* expression (*p* < 0.01) in PBLs. As the levels of WEAA added increased, the expression of *IKKβ*, *IκBα*, *NF-κBp65* and *IL-1β* exhibited a significant linear or quadratic increase (*p* < 0.05), whereas *IL-4* expression showed a significant linear increase (*p* < 0.01) or quadratic increasing trend (*p* = 0.057) in PBLs.

### 3.6. Immune Indices and mRNA Expression in Culture Medium of PDTC-Blocked PBLs

Table 3 showed the effects of WEAA on the level of immune indices in the culture medium of PDTC-blocked PBLs. The results of the main effect analysis indicated that PDTC significantly reduced the IgG, IgM, IL-1β, IL-2, and IL-4 levels in the PBLs culture medium (*p* < 0.01); conversely, WEAA noticeably elevated the levels of IgG, IL-4 (*p* < 0.01) and IL-6 (*p* < 0.05) in the PBLs culture medium, and showed a tendency to increase the content of IL-1β (*p* = 0.094). The interaction effect analysis results indicated that the interaction of WEAA and PDTC on the levels of IL-1β and IL-4 in the PBLs culture medium was significant (*p* < 0.05; *p* < 0.01) and that the interaction effect on the expression of IL-2 in the PBLs showed a significant tendency (*p* = 0.054). In comparison to the control group, the PDTC(−)/WEAA(+) group showed an increase in the levels of IgG, IL-1β, IL-2 and IL-4 (*p* < 0.05) in PBLs culture medium; the PDTC(+)/WEAA(−) group decreased the IgM, IL-1β, and IL-6 content (*p* < 0.05), and the PDTC(+)/WEAA(+) group decreased the IgM and IL-1β content (*p* < 0.05) in PBLs culture medium.

**Figure 6:**
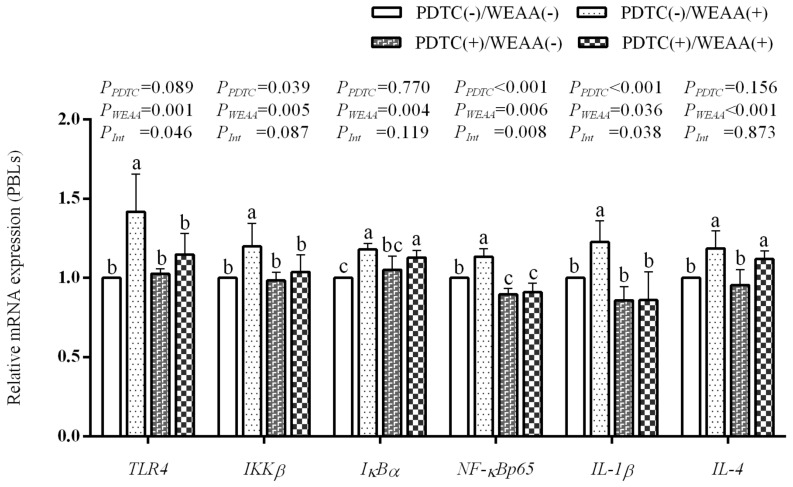
Effect of WEAA levels on the expression of TLR4/NF-κB signaling pathway-related genes in culture medium of PDTC-blocked PBLs. Abbreviations: TLR4 = Toll Like Receptor 4; IKKβ = Inhibitor of Nuclear Factor Kappa B Kinase Subunit Beta; IκB-α = NFKB Inhibitor Alpha; NFκBp65 = RELA Proto-Oncogene; IL-1β = Interleukin 1β; IL-4 = Interleukin 4; PDTC = Pyrrolidine Dithiocarbamate (NFκB inhibitor); WEAA = Water Extracts of *Artemisia annua* L. (100 µg/mL), values were expressed as the means of 6 replicates in each group. WEAA×PDTC = Interaction between WEAA and PDTC; WEAA = Effect of WEAA alone; PDTC = Effect of PDTC alone. Values within a row with different superscripts (a, b, c) differ significantly at *p* ≤ 0.05, whereas the probability value of 0.05 < *p* < 0.10 was considered as a tendency.

In Figure 6, the results of the main effect analysis indicate that PDTC significantly reduced the gene expression of *NF-κBp65*, *IKKβ* and *IL-1β* (*p* < 0.01), and tended to reduce the expression of *TLR4* (*p* = 0.069) in the PBLs; however, WEAA significantly increased *TLR4*, *IKKβ*, *IκBα*, *NF-κBp65*, *IL-4* expression (*p* < 0.01), and *IL-1β* (*p* < 0.05) expression in the PBLs. The results of the interaction effect analysis revealed that the interaction effect of WEAA and PDTC on the gene expression of *TLR4*, *NF-κBp65* and *IL-1β* was significant (*p* < 0.05; *p* < 0.01; *p* < 0.05) and that the interaction effect on the expression of *IKKβ* in the PBLs showed a significant tendency (*p* =0.087). In comparison to the control group, the PDTC(−)/WEAA(+) group showed an increase in the expression of *TLR4*, *IκBα*, *NF-κBp65*, *IL-1β* and *IL-4* in PBLs (*p* < 0.05); the PDTC(+)/WEAA(−) group decreased the expression of *NF-κBp65* (*p* < 0.05), and the PDTC(+)/WEAA(+) group increased the expression of *IκBα* and *IL-4* (*p* < 0.05) and decreased the expression of *NF-κBp65* (*p* < 0.05) in PBLs.

## 4. Discussion

*A. annua* is an extensively used botanical species in traditional pharmacology, whose antimalarial efficacy has undergone long-term clinical validation in traditional Chinese medicine. Contemporary investigations of *A. annua* and its bioactive compounds have demonstrated multifaceted application potential in animal husbandry, including antiparasitic properties, anti-inflammatory and immunomodulatory functions, and growth performance enhancement. These findings provide a new direction for its application in livestock and poultry breeding [42,43,44,45]. In the field of regulating livestock and poultry growth performance, the positive effects of extracts from *Artemisia* (Asteraceae family) have been verified by multiple studies. Research has demonstrated that *A. argyi* extract could significantly reduce the F/G in sheep, indicating a reduction in feed requirements per unit of weight gain and directly reflecting enhanced feed conversion efficiency [35]. Similarly, Gholamrezaie et al. [46] observed that dietary supplementation with *A. annua* extract increased ADG while significantly decreasing ADFI and F/G in broiler chickens, thereby providing further evidence for the growth performance-improving effects in animals. This effect in monogastric animals primarily relies on enhanced intestinal digestive enzyme activity and anti-inflammatory responses, with regulatory targets focused on hindgut digestion and immune barrier function. In contrast, ruminants exhibit a distinct regulatory pattern centered on rumen microbial fermentation. For instance, Faryabi et al. (2023) [47] reported that replacing alfalfa hay with *A*. *sieberi* (rich in flavonoids and terpenoid compounds) in lamb diets did not significantly affect ADG or F/G, but markedly reduced ADFI. The core mechanism is that secondary metabolites can regulate rumen microbial community structure, improve dry matter and fibrous nutrient digestibility, and enhance energy conversion efficiency by optimizing the acetate-to-propionate ratio, ultimately maintaining the homeostasis of body metabolism and health. In the present study, the effects of dietary WEAA supplementation on sheep were consistent with the aforementioned findings regarding *Artemisia* extracts. WEAA exerted no significant effect on sheep ADG but significantly decreased ADFI, suggesting that sheep can maintain normal growth rates with lower feed intake and indirectly indicating that WEAA may improve feed utilization efficiency to ensure adequate nutrient acquisition from limited feed resources. Previous studies have confirmed that plant bioactive components can improve nutrient utilization efficiency in animals by regulating gastrointestinal microecology and digestive functions [35,47]. Based on this, we hypothesize that the growth-regulating effect of WEAA is also closely associated with its abundant bioactive constituents, including organic acids, polysaccharides, flavonoids, and terpenoids. These constituents may optimize growth performance by promoting digestive enzyme activity in the gastrointestinal tract of sheep, improving mucosal absorption capacity, or modulating gastrointestinal microbiota composition, thereby enhancing the digestive and absorptive efficiency of feed nutrients.

To systematically elucidate the immunomodulatory mechanisms of WEAA and determine its appropriate application dose, this study combined in vitro and in vivo experiments for investigations. Previously, Zhao et al. (2024) [18] performed compositional analysis of WEAA utilizing ultra-high-performance liquid chromatography, identifying substantial enrichment in terpenoid compounds. Concurrent cytotoxicity assessments via MTT assay demonstrated that WEAA concentrations ranging from 31.3 μg/mL to 250 μg/mL exhibited negligible toxicity toward primary dermal keratinocytes, allowing normal cellular proliferation, whereas excessive concentrations of 880 μg/mL induced cytotoxic effects. Zhang et al. (2022) [31] isolated three water-soluble polysaccharides from *A. annua* (including β-linked polysaccharides containing pyranose and β-fructofuranosides), demonstrating that these polysaccharides effectively inhibit TNF-α and IL-6 production in murine macrophages. Notably, at a concentration of 125 μg/mL, cellular viability remained elevated without manifestation of overt toxicity, indicating that *A. annua* polysaccharides possess immunomodulatory potential with favorable biosafety profiles within this concentration range. Furthermore, Wan et al. (2016) [48] corroborated that elevated levels of phenolic compounds (44.24 ± 2.12 mg GAE/g) and flavonoid (2780 ± 2.25 mg RE/g) in *A. annua* foliage enhanced liver antioxidant capacity and immune competence in broiler chickens, consequently improving growth performance parameters. Our analytical investigations similarly identified the principal bioactive constituents of WEAA as organic acids, polysaccharides, flavonoids, and terpenoid compounds, and multiple studies have substantiated the immunomodulatory and antioxidant potential of these bioactive substances. For example, Lan et al. (2010) [49] confirmed that *A*. *argyi* polysaccharides promoted splenic lymphocyte proliferation in a dose-dependent manner and could promote ConA-induced secretion of IFN-γ and IL-2 by splenic cells, suggesting that Th1 cells may be the primary target cells for the immunomodulatory effects of *A*. *argyi* polysaccharides. Du et al. (2023) [13] further confirmed that *A*. *ordosica* polysaccharides, by targeting the TLR4 receptor, significantly upregulated mRNA expression levels of key genes (*TLR4*, *MyD88*, *P38 MAPK*, *JNK*, and *NF-κB p50*) in the TLR4/MAPK/NF-κB signaling pathway in spleen and intestinal tissues, thereby driving the secretion of immunoglobulins (IgA, IgG, IgM) and cytokines (IL-1β, IL-2, IL-4). Additionally, Niu et al. (2020) [50] showed that enzymatically treated *A*. *annua* (rich in flavonoids and polyphenols) increased piglet IL-10, sIgA, and IgG levels in the ileum, enhancing mucosal immunity; terpenoid compounds can also participate in immune signal transduction through the TLR4/NF-κB pathway [51]. Although the present investigation has not yet conducted standardized characterization of WEAA active constituents, systematic bioavailability assessments, or comprehensive toxicological evaluations, the aforementioned research findings provide empirical foundations for the safe application and compositional characterization of WEAA. These results suggested that subsequent research should focus on isolating and identifying critical bioactive components, elucidating synergistic interaction mechanisms, and conducting systematic toxicological assessments to facilitate standardized applications in livestock production systems.

To elucidate the impact of WEAA on ovine immunological function, our experimental protocol involved dietary supplementation with graduated dosages (0, 500, 1000, and 1500 mg/kg) of WEAA. The results demonstrated a dose-dependent increase in IgA and IgG concentrations (key indicators of humoral immunity), as well as elevated levels of IL-1β, IL-2, and IL-4 (core immunoactive cytokines) in serum, liver, and spleen tissues, with the 1000 mg/kg treatment group exhibiting optimal efficacy. Notably, the pleiotropic pro-inflammatory cytokine IL-1β was elevated alongside coordinated upregulation of IL-2 and IL-4 in this study, with no isolated pro-inflammatory hyperactivity: 1000 mg/kg WEAA upregulated spleen IL-1β, liver IL-2, and serum and liver IL-4; 1500 mg/kg WEAA increased serum IL-1β while maintaining IL-2 and IL-4 balance and preventing pro-inflammatory overactivation. This coordinated cytokine pattern supports WEAA-mediated IL-1β elevation as a form of physiological immune activation [13,31]. These findings are consistent with the documented immunomodulatory effects of *A. annua* and its bioactive constituents (terpenoid compounds, polysaccharides, flavonoids, and essential oils) observed in broiler chickens, mice, piglets, and aquatic species [46,50,52,53], corroborating the sophisticated regulatory paradigm by which natural products activate systemic and localized immune responses. Earlier results of this study revealed that WEAA improved the rumen immune function of sheep by increasing ruminal sIgA, IL-4, and IL-2 concentrations while upregulating corresponding gene expression. Concurrently, WEAA enhanced the abundance of beneficial ruminal microorganisms, including *g_Lachnospiraceae_NK3A20_group*, *g_Saccharofermentans*, *g_Marvinbryantia*, and *g_Bifidobacterium* species, establishing a regulatory pathway linking ruminal microecology, localized immunity, and systemic immunity [41]. These results were consistent with those of Li et al. (2023) [36], demonstrating that dietary supplementation with *A. ordosica* polysaccharides increased serum IL-1β and IL-6 concentrations while enhancing the abundance of beneficial ruminal microflora (*norank_f_F082, Lachnospiraceae_FE2018_group*) in goats. Their research confirmed that mild oxidative imbalance facilitates stress-defense autoregulation in organisms via elevated pro-inflammatory mediators. Based on the above results and related studies, WEAA’s immunomodulatory effects arise from the synergy of its multiple bioactive components: polysaccharides activate intracellular signaling via immune cell surface receptors (e.g., TLR2/4) to directly boost antibody and cytokine secretion, enhancing humoral and cellular immunity; terpenoids and flavonoids regulate immune pathways to strengthen responses and maintain inflammatory balance, which is consistent with the findings of Song et al. (2022) [54], who found that the ethanol extract of *A. annua* (containing phenolic acids, flavonoid, terpenoids, and coumarins) attenuated LPS-induced hyperactivation of TNF-α, IL-1β, and IL-6 in bovine mammary epithelial cells; and organic acids lower rumen pH to facilitate beneficial microbial colonization and enhance intestinal mucosal barrier function, thereby indirectly improving systemic immunity [55,56,57]. It should be noted that the absence of a control pair-feeding in this study means potential interference from reduced ADFI on organism physiological status cannot be excluded. Thus, the observed changes in immune indicators cannot be fully attributed to the direct regulatory effect of WEAA. This is a limitation in the analysis of the immune regulatory mechanism of WEAA, which urgently needs to be verified by targeted experiments in subsequent studies.

To further elucidate the molecular mechanisms underlying WEAA-mediated immunological regulation in sheep, this investigation focused on the TLR4/NF-κB signaling cascade—a key immune response regulatory pathway. The results demonstrated that dietary WEAA supplementation significantly upregulated mRNA expression levels of upstream TLR4/NF-κB signaling molecules (*TLR4*, *IKKβ*, *IκBα*) and the core transcriptional factor *NF-κBp65* in the liver and spleen of sheep. These findings indicate that WEAA initiates pathway activation by enhancing TLR4-mediated immune signal recognition, then promotes TLR4/NF-κB cascade activation via upregulating *IKKβ* and *NF-κB p65* expression and regulating *IκBα* metabolism. Concurrently, WEAA significantly upregulated liver mRNA expression levels of *IL-1β* (innate immunity initiator) and *IL-4* (humoral immunity mediator), as well as spleen *IL-4* mRNA expression, consistent with prior measurements of IL-1β and IL-4 content. These results confirm that the TLR4/NF-κB pathway is likely the pivotal molecular mechanism by which WEAA modulates ovine immune indicators, achieving coordinated regulation of humoral and innate immunity. Extensive research demonstrates that *Artemisia* species and their extracts exert beneficial bidirectional modulatory in both challenge models and conventional feeding trials, providing crucial insights into their functional characteristics across diverse physiological states [12,36]. For instance, investigations have shown that dietary *A. ordosica* polysaccharide supplementation significantly upregulates jejunal *MyD88*, *IL-1β*, and *IL-6* mRNA expression levels through TLR4-MyD88-NF-κB pathway activation, improving growth performance while substantially modulating intestinal immunity and preventing enteric inflammation [58]. Yang et al. (2020) [59] confirmed that *A. rupestris* aqueous extracts, with polysaccharides as primary bioactive constituents, interacted with TLR2/TLR4 receptors on immune cell membranes, activating MAPKs and NF-κB signaling pathways. This leads to dose-dependent upregulation of *IL-1β* and *IL-6* mRNA expression in dendritic cells, thereby activating cellular immunity. Additionally, Zhang et al. (2018) [60] further clarified the immunomodulatory mechanisms of *A. annua* polysaccharides, showing that they effectively activate RAW 264.7 macrophage secreting IL-6 and TNF-α, with strong immunoactivation potential and minimal cytotoxicity. In conclusion, WEAA likely orchestrates synergistic regulation of ovine innate and humoral immunity through TLR4/NF-κB pathway activation.

Nevertheless, further in vitro experiments are required to confirm whether WEAA’s immunomodulatory effects depend on TLR4/NF-κB pathway mediation. PBLs are core circulating immune cells, including T cells, B cells, and natural killer cells that coordinately regulate cellular immunity (e.g., T cell activation, cytokine secretion) and humoral immunity (e.g., B cell differentiation, antibody production) [32,61]. Their functions align with immune cells in key organismal tissues, regulating targeted cytokines (IL-1β, IL-2) and antibodies (IgA, IgG), and thus can reflect WEAA’s systemic immune regulatory effect in sheep. Furthermore, PBLs are commonly used in cell culture systems to investigate immune responses and related processes [62,63]. For instance, Yu et al. (2020) [64] showed that *Cyclocarya paliurus* polysaccharides (25–100 μg/mL) significantly enhanced murine spleen lymphocytes proliferation, in vitro stimulation of these lymphocytes also increased IL-2 and TNF-α secretion, thereby boosting murine cellular immune function. Prior studies have confirmed that *A. argyi* extracts promote T and B lymphocyte proliferation [49,65]. Our preliminary research has confirmed that the principal bioactive constituents of WEAA are polysaccharides, flavonoids, and terpenoid compounds. Based on this foundation, we further investigated WEAA’s effects on peripheral lymphocytes of sheep. Experimental results showed that adding WEAA to the culture medium of normally cultured PBLs enhanced PBLs viability, with the 100 μg/mL treatment group exhibiting optimal efficacy. Moreover, 50–200 μg/mL differentially enhanced the levels of IgA, IgG, IgM, IL-1β, and IL-2 in the culture medium of PBLs, while upregulating gene expression levels of TLR4/NF-κB signaling pathway-related factors in PBLs. We postulate that under physiological conditions, WEAA may bind to surface TLR4 receptors on PBLs, activating downstream signaling cascades, initiating immune gene transcription, and thereby exerting distinct immunomodulatory effects.

**Figure 7:**
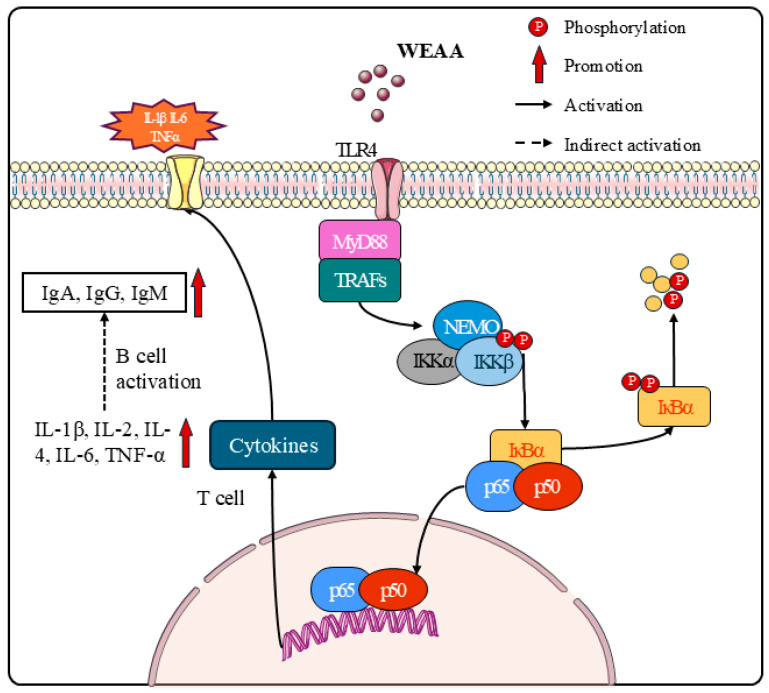
The schematic of the TLR4/NFκB signaling pathway modulated by WEAA.

To elucidate this mechanism, we selected the 100 μg/mL WEAA treatment group and used the NF-κB-specific inhibitor PDTC to suppress NF-κB activity. By measuring immunoglobulin content, cytokine secretion, and related gene expression in sheep lymphocytes, we aimed to further verify whether WEAA’s immunomodulatory effects on these cells are mediated via the TLR4/NF-κB pathway. By comparing differential responses across treatment groups, we aimed to establish the intrinsic link between WEAA’s immunomodulatory effects on sheep lymphocytes and the TLR4/NF-κB pathway. Our results showed that compared to controls, WEAA supplementation increased IgG, IgM, IL-1β, IL-2, and IL-4 levels in PBL culture supernatants, while concurrently upregulating TLR4/NF-κB pathway-related gene expression. Furthermore, PDTC is a canonical NF-κB inhibitor, while NF-κB functions as a transcription factor regulating cellular immunity and inflammatory responses [20,40]. Research demonstrates that PDTC acts via two primary mechanisms: interfering with cell surface receptor signal transduction to block upstream NF-κB activation pathways [66] and enhancing IκB synthesis to inhibit its phosphorylation and degradation, thereby retaining NF-κB in an inactive NF-κB-IκB complex [27]. Our experimental data indicated that PDTC alone or combined with WEAA significantly reduced IL-1β levels and *NF-κBp65* expression in PBLs. This suggests that PDTC inhibits NF-κB activation by preserving IκBα-NF-κB binding and potentially suppressing *NF-κBp65* phosphorylation [66], while IL-1β downregulation—as a key downstream mediator—confirms PDTC’s action through the NF-κB pathway. Additionally, WEAA exerted no significant effects when co-administered with PDTC, which was potentially attributable to PDTC’s strong inhibitory effects masking WEAA’s potential or opposing effects on the same signaling pathways with PDTC dominating. In contrast, *IL-4* expression remained consistent with WEAA supplementation, potentially due to IL-4 regulation by other signaling pathways, and the specific pathways involved and their crosstalk with the TLR4/NF-κB pathway await further investigation. Consequently, we speculate that WEAA enhances PBL immune function by activating the TLR4/NF-κB pathway, thereby upregulating expression of *TLR4*, *NF-κB*, and their downstream target genes (Figure 7). While this study preliminarily clarified WEAA’s immune mechanism in sheep via TLR4/NF-κB pathway analysis, it has limitations: pathway activation was only validated at the mRNA level, with no complementary protein-level assays (e.g., Western blot for NF-κBp65 phosphorylation and TLR4 expression). This has hindered the complete characterization of the pathway’s translational and post-translational modification regulatory features. Additionally, the present study did not perform targeted blocking experiments using TLR4-specific inhibitors (e.g., TAK-242), which prevented direct confirmation of TLR4’s core mediating role in the pathway. Therefore, future studies should supplement protein-level validation and TLR4-targeted inhibition experiments to further enhance the rigor and persuasiveness of the mechanism interpretation.

## 5. Conclusions

In conclusion, WEAA demonstrated significant immunomodulatory functions. Both in vitro and in vivo investigations indicated that WEAA might regulate the TLR4/NF-κB signaling pathway to upregulate the expression of *TLR4*, *IKKβ*, *IκBα*, *NF-κBp65*, as well as *IL-1β* and *IL-4* genes in liver and spleen. Furthermore, WEAA exhibited dose-dependent efficacy in significantly elevating the concentrations of immunoglobulins (IgA, IgG) and cytokines (IL-1β, IL-2, IL-4) in serum, liver and spleen, thereby enhancing both humoral and innate immune responses in sheep, with the optimal effect observed at a dietary supplementation level of 1000 mg/kg. These findings substantiate WEAA as a promising natural feed additive with considerable potential for application in sheep husbandry. Nevertheless, further investigation is required to elucidate the precise molecular mechanisms through which individual bioactive constituents within WEAA contribute to these observed immunoenhancing effects.

## Figures and Tables

**Table 1:** Effects of dietary WEAA on growth performance of meat-type sheep.

Item	WEAA Supplemental Level, mg/kg ^1^				SEM	*p*-Value ^2^	
	0	500	1000	1500		Linear	Quadratic
d 1–30							
ADG, g/d	170.95	187.14	190.83	176.25	9.90	0.852	0.751
ADFI, g/d	1608.28 ^a^	1390.79 ^ab^	1338.63 ^b^	1365.33 ^ab^	38.57	0.025	0.024
F:G, g/g	10.71 ^a^	7.72 ^b^	7.26 ^b^	7.75 ^b^	0.49	0.047	0.030
d 30–60							
ADG, g/d	214.29	208.75	210.95	203.89	9.37	0.767	0.958
ADFI, g/d	1714.89 ^a^	1502.92 ^b^	1439.81 ^b^	1526.29 ^ab^	38.20	0.065	0.025
F:G, g/g	8.01	7.26	6.90	7.74	0.25	0.663	0.342
d 1–60							
ADG, g/d	192.62	189.72	196.90	187.86	6.45	0.907	0.967
ADFI, g/d	1661.58 ^a^	1467.40 ^b^	1430.40 ^b^	1478.18 ^b^	33.75	0.050	0.028
F:G, g/g	8.89 ^a^	7.58 ^ab^	7.27 ^b^	7.88 ^ab^	0.23	0.147	0.050

Abbreviations: ADG = Average Daily Gain; ADFI = Average Daily Feed Intake; F:G = Feed to Gain Ratio; SEM = Standard Error of the Mean. ^1^ WEAA = Water Extracts of *Artemisia annua* L.; Different concentrations of WEAA (0, 500, 1000, and 1500 mg/mL), values were expressed as the means of 8 sheep in each group. ^2^ Assesses the linear and quadratic effects on various response indices as a function of increasing WEAA supplementation levels. Values within a row with different superscripts (a, b) differ significantly at *p* ≤ 0.05, whereas the probability value of 0.05 < *p* < 0.10 was considered as a tendency.

**Table 2:** Effects of dietary WEAA on the immune indexes in culture medium of PBLs.

Item	WEAA Supplemental Level, µg/mL ^1^						SEM	*p*-Value ^2^	
	0	25	50	100	200	400		Linear	Quadratic
IgA, μg/mL	6.28 ^b^	5.86 ^b^	8.13 ^a^	8.30 ^a^	6.29 ^b^	5.72 ^b^	0.34	0.123	0.041
IgG, μg/mL	13.92 ^e^	14.51 ^de^	15.13 ^cd^	17.77 ^a^	16.09 ^b^	15.42 ^bc^	0.28	0.208	<0.001
IgM, μg/mL	11.7 ^b^	11.77 ^b^	11.58 ^b^	12.76 ^ab^	13.6 ^a^	11.84 ^b^	0.23	0.758	0.052
IL-1β, pg/mL	78.76 ^c^	79.57 ^c^	83.35 ^bc^	92.86 ^a^	87.72 ^b^	87.12 ^b^	1.15	0.028	<0.001
IL-2, pg/mL	115.31 ^c^	131.71 ^abc^	148.26 ^a^	137.77 ^ab^	140.62 ^ab^	124.35 ^bc^	3.50	0.784	0.034
IL-4, pg/mL	51.42 ^c^	58.26 ^bc^	64.87 ^ab^	69.79 ^a^	65.26 ^ab^	65.34 ^ab^	1.78	0.053	0.002
IL-6, pg/mL	65.71	59.94	60.97	61.31	61.87	62.67	0.84	0.990	0.660

Abbreviations: IgA = Immunoglobulin A; IgG = Immunoglobulin G; IgM = Immunoglobulin M; IL-1β = Interleukin 1β; IL-2 = Interleukin 2; IL-4 = Interleukin 4; IL-6 = Interleukin 6; SEM = Standard Error of the Mean. ^1^ WEAA = Water Extracts of *Artemisia annua* L.; Different concentrations of WEAA (0, 25, 50, 100, 200 and 400 µg/mL), values were expressed as the means of 6 replicates in each group. ^2^ Assesses the linear and quadratic effects on various response indices as a function of increasing WEAA supplementation levels. Values within a row with different superscripts (a, b, c, d, e) differ significantly at *p* ≤ 0.05, whereas the probability value of 0.05 < *p* < 0.10 was considered as a tendency.

**Table 3:** Effects of WEAA on immune indices in culture medium of PDTC-blocked PBLs.

Item	PDTC (−) ^1^		PDTC (+)		SEM	*p*-Value ^3^		
	WEAA (−) ^2^	WEAA (+)	WEAA (−)	WEAA (+)		PDTC	WEAA	WEAA × PDTC
IgA, μg/mg	6.21	6.83	6.12	5.79	0.17	0.096	0.660	0.155
IgG, μg/mg	19.89 ^b^	22.47 ^a^	17.96 ^b^	20.04 ^b^	1.48	0.007	0.004	0.740
IgM, μg/mg	10.18 ^a^	11.21 ^a^	7.38 ^b^	7.84 ^b^	0.46	<0.001	0.281	0.678
IL-1β, pg/mg	48.47 ^b^	64.36 ^a^	34.34 ^c^	32.80 ^c^	3.67	0.003	0.094	0.019
IL-2, pg/mg	106.80 ^b^	115.15 ^a^	97.32 ^b^	98.37 ^b^	2.49	0.007	0.425	0.054
IL-4, pg/mg	31.80 ^b^	50.42 ^a^	25.44 ^b^	26.91 ^b^	2.55	<0.001	0.003	0.009
IL-6, pg/mg	42.25 ^ab^	48.18 ^a^	39.52 ^b^	45.50 ^ab^	1.30	0.269	0.021	0.993

Abbreviations: IgA = Immunoglobulin A; IgG = Immunoglobulin G; IgM = Immunoglobulin M; IL-1β = Interleukin 1β; IL-2 = Interleukin 2; IL-4 = Interleukin 4; IL-6 = Interleukin 6; SEM = Standard Error of the Mean. ^1^ PDTC = Pyrrolidine Dithiocarbamate, NFκB inhibitor. ^2^ WEAA = Water Extracts of *Artemisia annua* L. (100 µg/mL); values were expressed as the means of 6 replicates in each group. ^3^ WEAA × PDTC = Interaction between WEAA and PDTC; WEAA = Effect of WEAA alone; PDTC = Effect of PDTC alone. Values within a row with different superscripts (a, b, c) differ significantly at *p* ≤ 0.05, whereas the probability value of 0.05 < *p* < 0.10 was considered as a tendency.

## Data Availability

The original contributions presented in this study are included in the article/Supplementary Materials. Further inquiries can be directed to the corresponding author(s).

## Supplementary Materials

The following supporting information can be downloaded at https://www.mdpi.com/article/10.3390/ani16010059/s1, Figure S1: Total ion current chromatogram of WEAA metabolites in negative ion mode (NEG); Figure S2: Total ion current chromatogram of WEAA metabolites in positive ion mode (POS); Table S1: Compound content of WEAA (DM basis, %); Table S2: Composition and nutrient levels of the basal diet (%, air-dry basis); Table S3: Primers for target genes used in qRT-PCR.

## Abbreviations

The following abbreviations are used in this manuscript:

IgA	Immunoglobulin A.
IgG	Immunoglobulin G.
IgM	Immunoglobulin M.
IL-1β	Interleukin-1β.
IL-2	Interleukin-2.
IL-4	Interleukin-4.
IL-6	Interleukin-6.
TLR4	Toll-like receptor 4.
MyD88	Myeloid differentiation primary response protein 88.
IKKβ	Inhibitor of kappa B kinase.
IκBα	IκB alpha.
NF-κBp65	Nuclear factor kappa light-chain enhancer of activated B cells p65 subunit.
