# Supplementary material for: Immunomodulatory Effect of Artemisia annua L. Water Extract on Meat-Type Sheep via Activating TLR4/NF-κB Signaling Pathways
Source: Animals (Basel). 2025 Dec 24;16(1):59. doi: 10.3390/ani16010059 (PMC12784896; doi:10.3390/ani16010059)
Supplement: Supplementary file 1 [file animals-16-00059-s001.zip › animals-4031675-supplementary.pdf]

Immunomodulatory Effect of *Artemisia annua* L. Water Extract on Meat-Type Sheep via Activating TLR4/NF-κB Signaling Pathways

Gen Gang, Ruiheng Gao, Shiwei Guo, Yu Xin, Xiao Jin, Yuanyuan Xing, Sumei Yan, Yuanqing Xu and Binlin Shi 1,*

Table S1

Compound contents of WEAA (DM basis,%)

Compounds	contents
Organic acids and derivatives	24.61
Soluble polysaccharide	18.64
Flavonoids	9.80
Prenol lipids	7.75
Organoheterocyclic compounds	7.75
Organooxygen compounds	5.01
Nucleosides, nucleotides, and analogues	5.01
Fatty acyls	4.79
Benzene and substituted derivatives	3.87
Glycerophospholipids	2.28
Coumarins and derivatives	2.05
Cinnamic acids and derivatives	1.82
Phenols	1.60
Others	5.01

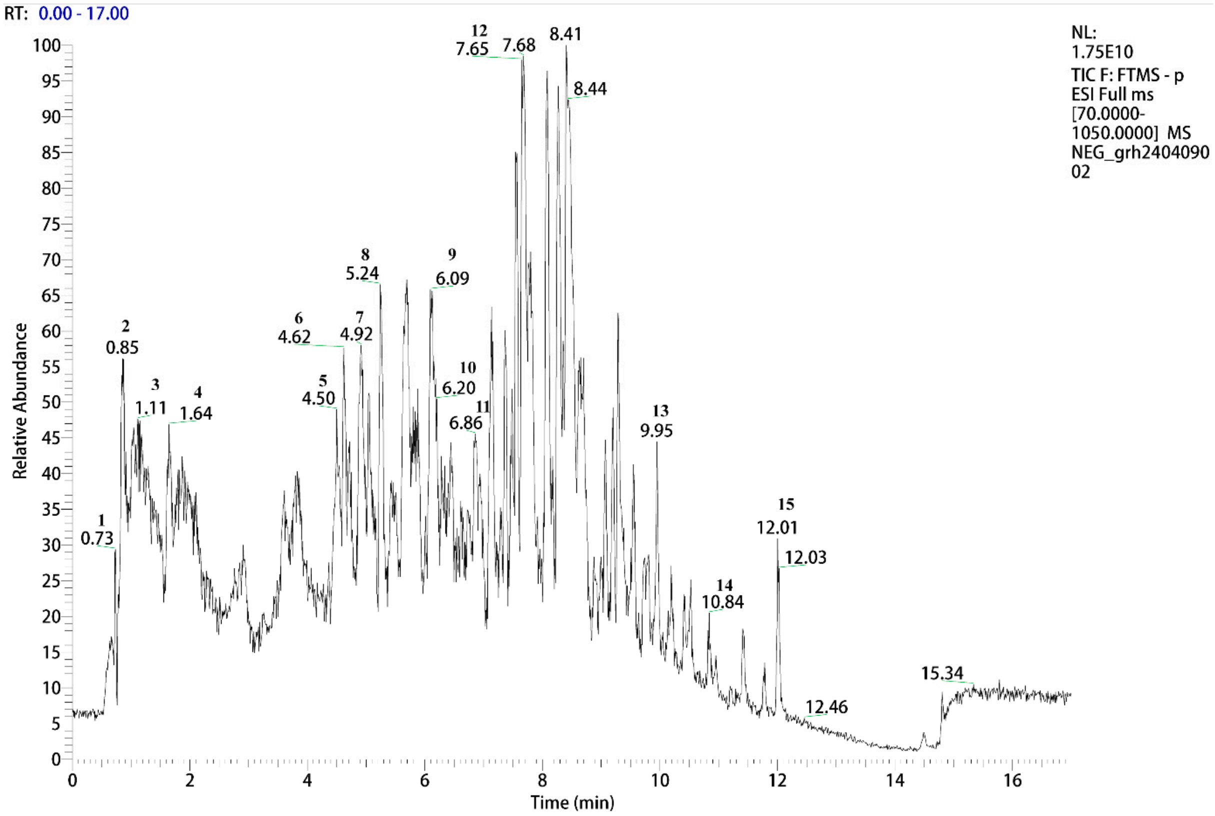

Figure S1 Total ion current chromatogram of WEAA metabolites in negative ion mode (NEG)

Note: 1. Myo-Inositol; 2. QUEBRACHITOL; 3. Unidentified peak; 4. Glucaric acid; 5. 3-O-Caffeoylquinic acid, 1-o-p-Coumaroyl-beta-d-glucose; 6. Cryptochlorogenic acid; 7. Palatinose; 8. Sucrose; 9. Isorhamnetin3-galactoside; 10. p-Hydroxybenzoic acid; 11. Melezitose; 12. (R)-2-Hydroxycaprylic acid; 13. Hydroxyvalerenic Acid; 14. Stearidonic acid; 15. Centaureidin

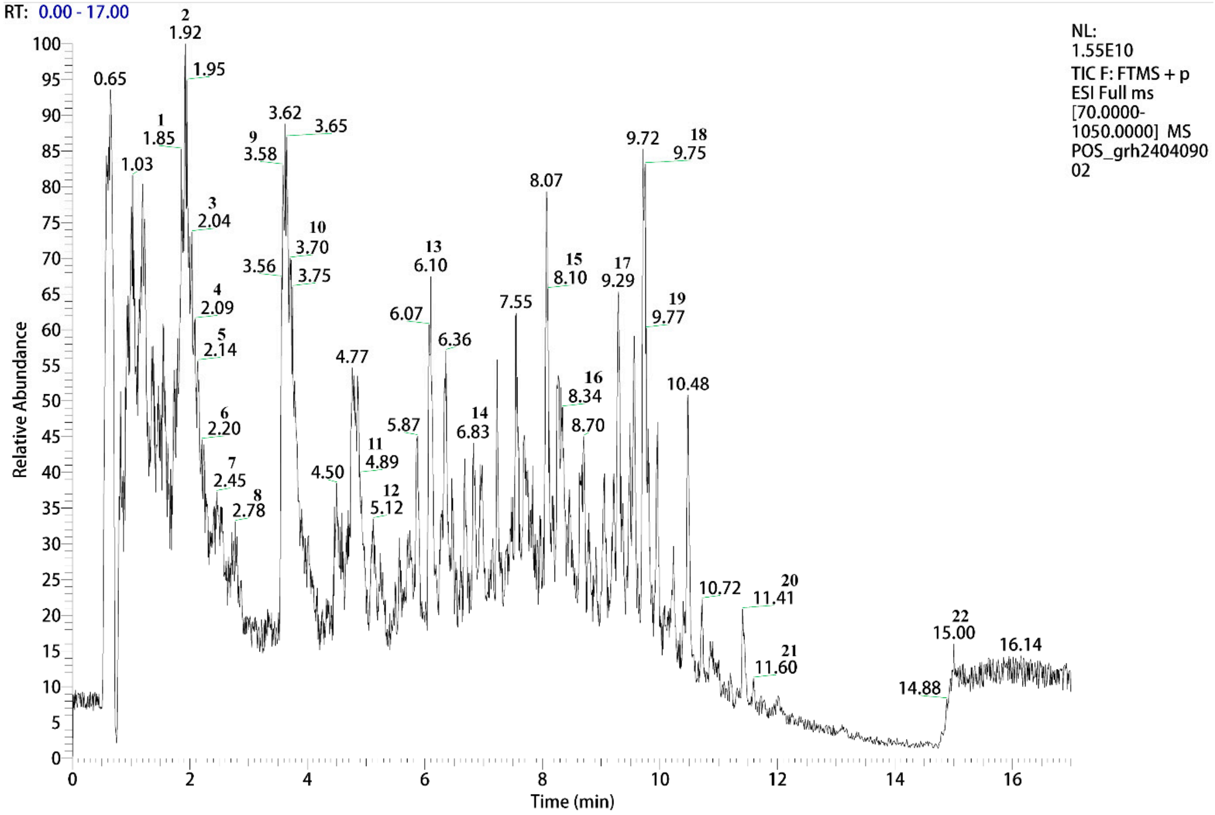

Figure S2 Total ion current chromatogram of WEAA metabolites in positive ion mode (POS)

Note: 1. Leucyl-Valine; 2. (E)-Cinnamic acid; 3. Glu Ser Leu, Val Ile Thr, Glu Gln Leu; 4. Ala Ser Ile, Gly Pro Val; 5. Pantothenic acid; 6. 4-hydroxy-3,5-dimethoxybenzoic acid; 7. Glu Thr Ile, Asp Thr Leu; 8. 1-O-(3-Hydroxy-4,5-dimethoxybenzoyl)hexopyranose; 9. Inosine; 10. Val Glu Val; 11. Asp Val Leu; 12. Laminine; 13. Phe Val Phe; 14. 15,16-Dihydrotanshinone I; 15. Aurantio-obtusin, 5,7,3'-Trihydroxy-6,4',5'-trimethoxyflavone, PC(14:0/0:0); 16. N-[(4-methoxyphenyl)methyl]-3-[1-[6-(propylamino)pyrimidin-4-yl]piperidin-4-yl]propenamide; 17. ISOLACTARORUFIN; 18. Arteannuin B; 19. Ilicic acid; 20. Isoalantolactone, 6Beta-Naltrexol; 21. Confertifoline; 22. Casticin

Table S2

Composition and nutrient levels of the basal diet (%, air-dry basis)

Item	Content
Ingredients	
Alfalfa	16.25
Corn straw grass	14.00
Oat grass	24.75
Corn	23.25
Soybean meal	10.95
Wheat bran	4.25
Corn germ meal	1.95
Soybean oil	1.10
Limestone	1.10
Calcium phosphate dibasic	0.70
Salt	0.40
Sodium bicarbonate	0.80
Premix [1]	0.50
Total	100.00
Nutrient levels [2]	
Digestible energy (MJ/kg)	12.01
Dry matter	89.89
Crude protein	15.80
Neutral detergent fiber	40.80
Acid detergent fiber	26.24
Calcium	1.08
Phosphorus	0.40

[1] The premix provided the following nutrient content for one kilogram of diet: vitamin A, 6000 IU; vitamin D3, 2500 IU; vitamin E, 12.5 IU; vitamin K3, 31.8 mg; vitamin B1, 0.035; vitamin B2, 8.5 mg; vitamin B6, 0.9 mg; nicotinic acid, 22 mg; D-pantothenic acid, 17 mg; vitamin B12, 0.03 mg; biotin, 0.14 mg; folic acid, 1.5 mg; Fe, 0.04 g; Cu, 0.008 g; Zn ,0.05 g; Mn, 0.03 g; I ,0.3 mg; Se ,0.3 mg; Co, 0.25 mg.

[2] Digestible energy was a calculated value, while the others were measured value.

Table S3

Primers for target genes used in qRT-PCR

Gene [1]	Sequence (5'->3') [2]	GenBank No.	Length/bp
β-actin	F- ACAATGTGGCCGAGGACTTT<br>R- GCCGTGATGGCTGACCATTC	NM_001009784.3	278
GAPDH	F- TTATGACCACTGTCCACGCC<br>R- TCAGATCCACAACGGACACG	NM_001190390.1	216
TLR4	F- CCTTGCGTACAGGTTGTTCC<br>R- GTCCAGCATCTCGGTTGACA	NM_001135930.1	99
IKKβ	F- GCCGCCCATTACAAGCTGAA<br>R- CTGGAAGAACGGGAGGTTCC	XM_042241396.1	165
IκB-α	F- TCACCTACCAGGGCTACTCC<br>R- CTGTGAACTCTGATTCGGTGTC	NM_001166184.1	153
NF-κBp65	F- CTCCTCTCGGGGGATGAAGA<br>R- ATCCCTTGCTAACCCACTGC	XM_027959295.2	123
IL-1β	F- CTGTGGCCTTGGGTATCAGG<br>R- GCCACCTCTAAAACGTCCCA	NM_001009465.2	251
IL-4	F- GCTGAACATCCTCACATCGAG<br>R- TTCTCAGTTGCGTTCTTTGG	AF1721681	87

Abbreviations: β-actin= beta Actin; GAPDH= Glyceraldehyde-3-phosphate Dehydrogenase; TLR4 = Toll Like Receptor 4; IKKβ = Inhibitor of Nuclear Factor Kappa B Kinase Subunit Beta; IκB-α = NFKB Inhibitor Alpha; NFκBp65 = RELA Proto-Oncogene; IL-1β = Interleukin 1β; IL-4 = Interleukin 4; F= Forward Primer; R= Reverse Primer.
